# Characterization of sequence elements from Malvastrum yellow vein betasatellite regulating promoter activity and DNA replication

**DOI:** 10.1186/1743-422X-9-234

**Published:** 2012-10-11

**Authors:** Jie Zhang, Xinyue Zhang, Yaqin Wang, Huwei Hou, Yajuan Qian

**Affiliations:** 1 State Key Laboratory of Rice Biology, Institute of Biotechnology, Zhejiang University, Hangzhou 310029, People's Republic of China

**Keywords:** *Begomovirus*, Betasatellite, Malvastrum yellow vein virus, Promoter

## Abstract

**Background:**

Many monopartite begomoviruses are associated with betasatellites, but only several promoters from which were isolated and studied. In this study, the *βC1* promoter from Malvastrum yellow vein betasatellite (MYVB) was characterized and important sequence elements were identified to modulate promoter activity and replication of MYVB.

**Results:**

A 991 nucleotide (nt) fragment upstream of the translation start site of the *βC1* open reading frame of MYVB and a series of deletions within this fragment were constructed and fused to the β-glucuronidase (*GUS*) and green fluorescent protein (*GFP*) reporter genes, respectively. *Agrobacterium*-mediated transient expression assays showed that the 991 nt fragment was functional and that a 28 nt region (between −390 nt and −418 nt), which includes a 5′UTR Py-rich stretch motif, was important for promoter activity. Replication assays using *Nicotiana benthamiana* leaf discs and whole plants showed that deletion of the 5′UTR Py-rich stretch impaired viral satellite replication in the presence of the helper virus. Transgenic assays demonstrated that the 991 nt fragment conferred a constitutive expression pattern in transgenic tobacco plants and that a 214 nt fragment at the 3'-end of this sequence was sufficient to drive this expression pattern.

**Conclusion:**

Our results showed that the *βC1* promoter of MYVB displayed a constitutive expression pattern and a 5′UTR Py-rich stretch motif regulated both *βC1* promoter activity and MYVB replication.

## Background

The *Geminiviridae* are a family of plant DNA viruses whose members are classified into four genera: *Mastrevirus*, *Begomovirus*, *Curtovirus* and *Topocuvirus*. The majority of geminiviruses belong to the genus *Begomovirus*. Begomoviruses are either monopartite or bipartite in the organization of their genome and many monopartite begomoviruses are associated with betasatellites (formerly called DNAβ) [1-3]. Betasatellites depend on the helper begomoviruses for replication, encapsidation and insect transmission as well as spread within and between plants [2,4]. Comparison of the nucleotide sequences from all known betasatellite molecules reveals three conserved features: a highly conserved region called the satellite conserved region (SCR), a single gene (known as *βC1*) that is conserved in both position and size and is a determinant of symptoms, and an A-rich region [5-7].

Using *Agrobacterium*-mediated transient expression and stable transformation system, many motifs/sequences have been identified to be involved in regulation of geminivirus transcription [8-10]. For example, Shung et al. [11] identified two elements located upstream of AL1935 and AL1629, important for transcription of complementary sense RNAs derived from Tomato golden mosaic virus (TGMV). A region located between −125 nt and −60 nt from the transcription start site in the TGMV *CP* promoter was reported to be involved in both activation and derepression by TrAP [12,13]. However, few promoters from betasatellites have been isolated and studied since Guan and Zhou [14] first reported the characterization of the *βC1* promoter of the Tomato yellow leaf curl China betasatellite (TYLCCNB) and subsequently Eini et al. [10] identified sequence elements which regulated *βC1* transcription associated with the Cotton leaf curl Multan betasatellite (CLCuMB). Malvastrum yellow vein virus (MYVV) is a typical monopartite geminivirus. Previous reports have shown that the betasatellite associated with MYVV (MYVB) is involved in symptom induction and it is required for enhancing the accumulation of helper virus in tobacco plants [15]. In order to further elucidate the transcriptional regulation and replication of the MYVB, in this study, we have characterized the putative promoter of the *βC1* gene of MYVB using both transient and stable transgenic expression approaches. Furthermore, we have identified a motif consisting of a 5′UTR Py-rich stretch important for MYVB replication.

## Results

### Analysis of the putative promoter sequence of the MYVB *βC1* gene

**Figure 1:**
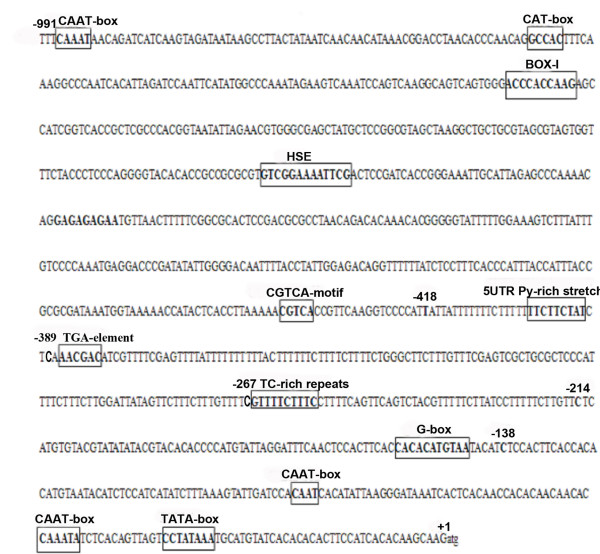
**Nucleotide sequence of the 991 nt fragment from the MYVB molecule.** The translation start site A is labeled +1. The position of the 5' deletion sites used to make promoter deletion constructs are indicated by individual characters above the sequence. All the putative motifs are shown in frame.

The sequence of the putative promoter encompassing the entire non-coding region (991 nt) upstream of the MYVB *βC1* open reading frame was analyzed using the PlantCARE program ( http://bioinformatics.psb.ugent.be/webtools/plantcare/html/). As illustrated in Figure 1, a number of putative regulatory motifs and *cis*-elements were predicted, including a typical TATA-box (−37 nt), some CAAT-boxes (−58 nt, −106 nt, −984 nt) and a G-box (−144 nt). Strikingly, compared with previously reported *βC1* promoters [10,14], the MYVB *βC1* promoter displayed key differences in the composition of the putative promoter. Of particular interest, was a 5′UTR Py-rich stretch, which usually plays an important role in increasing gene expression [16-18].

### Identification of *cis*-elements regulating *βC1* expression

**Figure 2:**
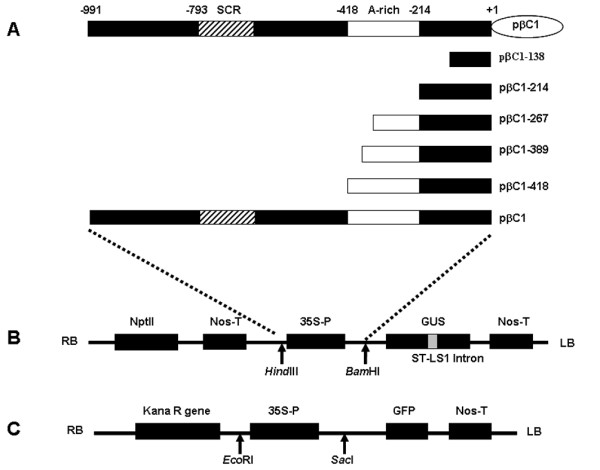
**Construction of different *βC1 *promoters of MYVB.** The satellite conserved region (SCR) and A-rich region are shown in boxes. The translation start site is numbered +1. (**A**) Schematic representation of the MYVB genome and various *βC1* promoters. (**B**) The promoters were cloned into the pINT121 binary vector. (**C**) The promoter constructs were cloned into the pCHF3:GFP vector.

**Figure 3:**
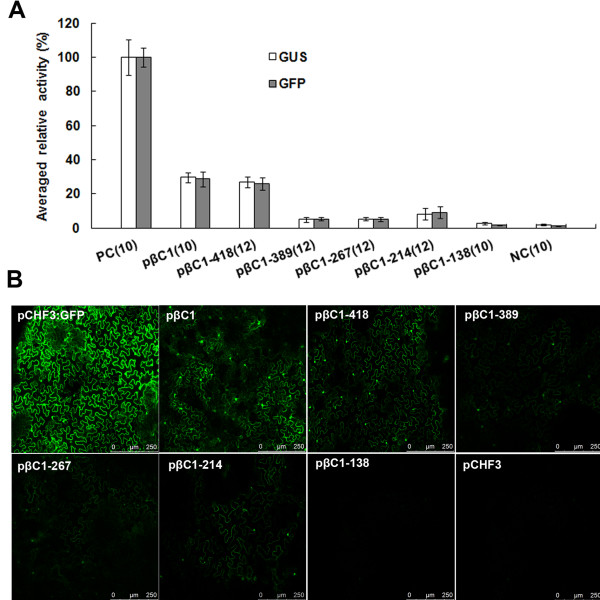
**Fluorometric activity analysis in *N *. *benthamiana *leaves after transient expression of pINT121 and pCHF3:GFP.** (**A**) The mean GUS or GFP activity from the CaMV 35S promoter of pINT121 or pCHF3:GFP was arbitrarily assigned as 100% and used to standardize the activity of all other constructs. The standard deviation of each construct is shown with error bars. The number of replicates of each sample is indicated in parentheses. PC, positive control, binary vector pINT121 or pCHF3:GFP; NC, negative control, promoter-less vector pBINGUS or pCHF3 vector without the *GFP* gene. (**B**) Fluorometric GFP activity analysis in *N*. *benthamiana* leaves after transient expression driven by various *βC1* promoter constructs. pCHF3:GFP and pCHF3 were used as positive and negative controls, respectively.

To determine the *cis*-elements responsible for the transcriptional control of MYVB *βC1*, the 991 nt fragment and a series of deletions within that sequence were constructed and fused to a promoter-less pINT121 vector (Figure 2). As a positive control, the pINT121 vector containing the *GUS* gene driven by the *Cauliflower mosaic virus* (CaMV) 35S promoter was used. Following *Agrobacterium*-mediated transient expression in tobacco leaves, fluorometric assays suggested that the promoter deletion constructs exhibited varying GUS expression levels (Figure 3A). The 991 nt fragment (pβC1) drove the greatest GUS expression that was approximately 29% of that observed in tissues infiltrated with the CaMV 35S promoter. Deletion of the region from −991 to −419 nt in the pβC1-418 construct resulted in only a minor difference in GUS expression levels compared with pβC1 (*P* > 0.05). Interestingly, deletion of the region from −991 to −390 nt (pβC1-389) resulted in a marked reduction in GUS expression levels to just 5% of that observed for the CaMV 35S promoter, while there were no significant differences among pβC1-389, pβC1-267 and pβC1-214 (*P* > 0.05). It is worth noting that deletion from −991 to −139 nt (pβC1-138) led to almost complete loss of GUS activity (Figure 3A).

In order to further identify the *cis*-elements involved in the transcriptional control of *βC1*, different promoter deletion sequences were inserted individually upstream of the *GFP* reporter gene within the expression vector pCHF3:GFP. The results revealed that 64 h after infiltration, significant differences in the intensity of GFP fluorescence were observed among the various constructs. As illustrated in Figure 3B, compared with other constructs, pβC1 and pβC1-418 produced relatively high levels of fluorescence, but much lower levels compared with the positive control pCHF3:GFP. GFP fluorescence was also observed to be produced from constructs pβC1-389 to pβC1-214, while the fluorescence of pβC1-138 was almost identical to that of the negative control pCHF3. Calculation of the fluorescence intensity revealed that the sequence within a 214 nt region upstream of the translation start site was fundamentally required for *βC1* promoter activity (Figure 3A). These results were consistent with those of the fluorometric GUS assay.

### A 5′UTR Py-rich stretch motif regulates *βC1* promoter activity

Figure 3 showed that deletion of the region from −991 to −390 nt in the pβC1-389 resulted in a remarkable reduction in promoter activity compared with pβC1-418, which indicated the presence of a positive *cis*-element in the region between −390 to −418 nt of the MYVB *βC1* promoter. Further sequence alignment analysis revealed the presence of a 5′UTR Py-rich stretch in this region. Therefore, the entire non-coding region promoter construct excluding the 5′UTR Py-rich stretch motif (pβC1ΔUTR) was obtained. Sixty-four hours after infiltration into leaves of *N*. *benthamiana* plants, fluorometric assays revealed that relative GUS activity of the pβC1ΔUTR declined to 11% of that driven by the CaMV 35S promoter, which differed significantly from that of pβC1 (*P* < 0.01).

### Roles of the 5′UTR Py-rich stretch motif in MYVB replication and pathogenicity

**Figure 4:**
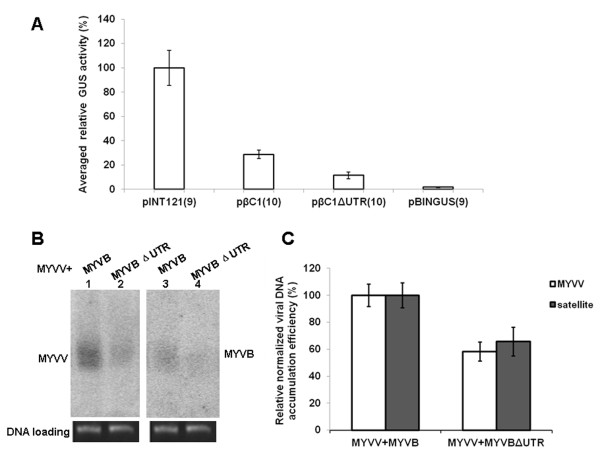
**The effect of the 5′UTR Py-rich stretch motif on MYVB promoter activity and MYVB replication.** (**A**) Fluorometric GUS activity analysis of the mutant promoter construct in *N*. *benthamiana* leaves. pINT121 and pBINGUS were used as positive and negative controls, respectively, and GUS activity of pINT121 was arbitrarily assigned as 100%. The number of replicates of each sample is indicated in parentheses. (**B**) Southern blot of total nucleic acid extracts of tobacco leaf co-inoculated with infectious clones of MYVV plus MYVB (lanes 1 and 3), or MYVV plus MYVBΔUTR (lanes 2 and 4). Blots were probed with either the *CP* gene sequence of MYVV (lanes 1 and 2) or the full-length MYVB sequence (lanes 3 and 4). Photographs of the ethidium bromide-stained gels are shown below the blot as an indication of loading control. (**C**) Densitometric quantification of band intensities. The value obtained from MYVV plus MYVB was arbitrarily assigned as 100%. Standard deviation values are based on three independent experiments.

To investigate the involvement of this 5′UTR Py-rich stretch motif in MYVB replication, the full-length MYVB sequence excluding the 5′UTR Py-rich stretch motif (MYVBΔUTR) and a tandem direct repeat of MYVBΔUTR were produced in the binary vector pBINPLUS. The infectious clone of MYVBΔUTR was tested for replication in *N*. *benthamiana* leaf discs assays using MYVV as the helper virus. Southern blot analysis showed that deletion of the 5′UTR Py-rich stretch motif resulted in a significant reduction in replication of both the betasatellite and helper virus (Figure 4B and C). The intensity value obtained from MYVV plus MYVB was arbitrarily assigned as 100%. The average normalized intensity values of blot bands are presented in Figure 4C. The results showed that deletion of the 5′UTR Py-rich stretch motif decreased viral DNA or betasatellite accumulation to 58% and 65%, respectively, compared with that of MYVV plus MYVB.

**Figure 5:**
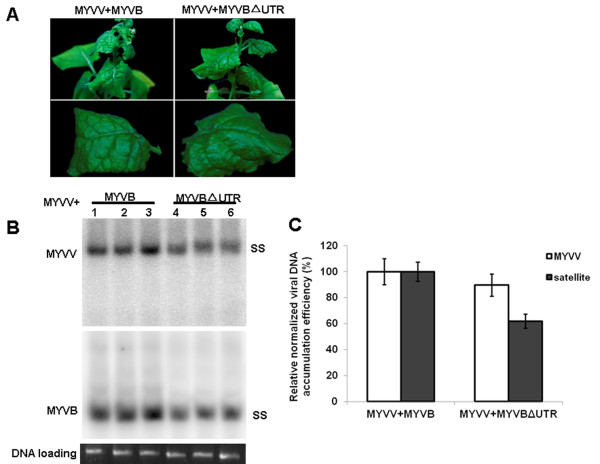
**Infectivity and symptoms induced by MYVV together with MYVB or MYVBΔUTR.** (**A**) Symptoms induced by infectious clones of MYVV plus MYVB or MYVBΔUTR on *N*. *benthamiana* plants at 30 dpi. (**B**) Southern blot analysis of viral and betasatellite DNAs in inoculated *N*. *benthamiana* plants. Nucleic acids were extracted from upper leaves of plants infected with MYVV and MYVB (lanes 1–3), MYVV and MYVBΔUTR (lanes 4–6). Blots were probed with the *CP* gene sequence of MYVV (top) or full-length of MYVB (bottom). The positions of single-stranded DNA (SS) forms are indicated. The lower panel represents an ethidium bromide-stained gel of DNA samples as a loading control. (**C**) Densitometric quantification of band intensities. The value obtained from MYVV plus MYVB was arbitrarily assigned as 100%. Standard deviation values are based on three independent experiments.

The roles of the 5′UTR Py-rich stretch motif in viral replication and symptom development were assayed in whole plants. Infectious clones of MYVBΔUTR or MYVB together with MYVV were inoculated into *N*. *benthamiana* plants. Both MYVBΔUTR and MYVB caused systemic infection in all plants tested, with similar symptoms including downward leaf curling as well as vein yellowing observed 30 days post-inoculation (dpi) (Figure 5A). The presence of viral DNA was determined by Southern hybridization analysis (Figure 5B and C). The results showed that the accumulation of the deleted MYVB was decreased to 62% compared with that of wild-type MYVB. Furthermore, a slight decrease in the accumulation of MYVV in tissues co-infected with MYVBΔUTR plus MYVV was observed, equivalent to 89% of that observed in tissues co-infected with MYVB plus MYVV (Figure 5B and C).

### Evaluation of the expression pattern in tobacco

**Figure 6:**
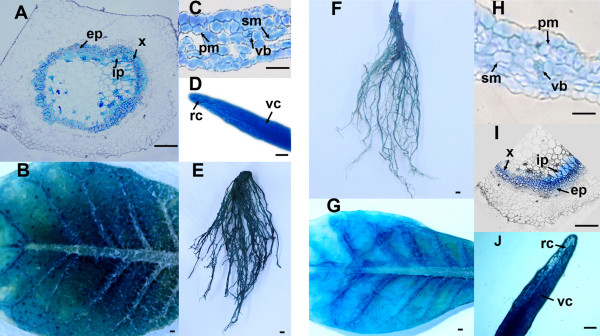
**Histochemical localization of GUS expression patterns of pβC1 (A-E) and pβC1-214 (F-J) in transgenic *N *.*tabacum *plants.** (**A**, **I**) Transverse sections of stem, (**B**, **G**) underside of the leaf, (**C**, **H**) transverse sections through the leaves, (**D**, **J**) longitudinal sections of the roots, and (**E**, **F**) root. Abbreviations: rc. root cap; vc. vascular cylinder; x. xylem; ph. phloem; ep. external phloem; ip. internal phloem; v. vein; pm. palisade mesophyll; sm. spongy mesophyll; vb. vascular bundle. Scale bars, 20 μm.

In order to determine the spatial expression pattern driven by the putative promoter from the MYVB *βC1* gene, the pβC1 and pβC1-214 constructs were introduced into tobacco plants via *Agrobacterium*-mediated transformation. Three independent transgenic lines of each construct with relatively high GUS activity were selected for histochemical analysis. In general, the transgenic pβC1 and pβC1-214 lines conferred the same expression pattern, with blue staining observed in almost all tissues of the roots, stems and leaves (Figure 6). In root sections prepared from pβC1 and pβC1-214 transgenic plants, GUS staining was observed both in the vascular cylinder and the root cap region (Figure 6D and J). In stem cross sections, GUS expression driven by pβC1 and pβC1-214 was observed mainly in parts of xylem parenchyma as well as internal and external phloem cells (Figure 6A and I). The GUS staining signal in pβC1 and pβC1-214 leaf sections was also found in different cell types, including palisade mesophyll cells, spongy mesophyll cells and the secondary vascular bundle (Figure 6C and H).

## Discussion

In this study, a 991 nt fragment upstream of the translation start site of *βC1* of MYVB was identified as the promoter and the 214 nt fragment from the 3′ end of the 991 nt fragment, which contains a G-box, was shown to be involved in basic promoter activity. Promoter activity was almost abolished in the 138 nt fragment from the 3′ end of the 991 nt without a G-box. These results suggested that the G-box acts as a positive regulatory element in the control of MYVB *βC1* transcription. Previous reports have indicated that G-box elements present in promoter regions of several geminiviruses and some plant genes bind to host factors involved in activating transcription [14,19]. Recently Eini et al. [10] also identified a 68 nt fragment containing a G-box upstream of the *βC1* gene associated with CLCuMB that was found to be important in the regulation of promoter activity. Furthermore, the G-box motif was shown to bind specifically to proteins in nuclear extracts from tobacco leaf tissues. We postulate that MYVB *βC1* shares a similar transcription regulation mechanism with other organisms although further investigations are required to elucidate the interaction of the MYVB G-box motif with host nuclear factors.

Previous evidence has shown that the 5′UTR Py-rich stretch motifs are highly transcription level-related sequence elements regulating the activity of various promoters [16-18]. As shown in Figure 4A, our results also demonstrated that site-directed deletion of the 5′UTR Py-rich stretch within the 991 nt *βC1* promoter sequence resulted in a 60% reduction in promoter activity compared with the intact *βC1* promoter, indicating the involvement of this element in the transcriptional regulation of *βC1*. In both fungi and animals, although transcription and DNA replication are divided into different biological processes, they frequently share the same regulatory elements [20-23]. Sequences/motifs involved in transcription or DNA replication have been detected in some geminiviruses [9,10,24-26]. Tu and Sunter [26] identified a conserved binding site within the TGMV AL-1629 promoter, which is necessary for efficient viral DNA replication. Previous studies that showed that betasatellites depend on the helper begomoviruses for replication [5,15]. However, up to date, the mechanism of interaction of begomovirus-encoded Rep with betasatellites to initiate satellite replication was not fully understood as well as betasatellites lack the iteron sequences encoded by their helper viruses. In this study, infectious assays in leaf discs showed that the 5′UTR Py-rich stretch motif also has an important role in MYVB replication.

Mutagenesis of the TYLCCNB and Tobacco Curly Shoot betasatellite (TbCSB) showed that the βC1 protein is the symptom determinant, although the promoter of *βC1* has some influence on symptom induction [27]. In our experiments, despite the production of low levels of betasatellite accumulation, the truncated MYVBΔUTR had no marked effect on the viral symptoms compared with the wild-type MYVB. Taken together, it is suspected that 5′UTR Py-rich stretch motif is involved in regulating the replication of betasatellite but is indispensable for viral symptom development.

Among these characterized geminivirus promoters, some are able to drive constitutive gene expression in transgenic plants, while others have more specific patterns of expression [8,14,28,29]. Histochemical staining assays revealed that the 991 nt fragment and the 214 nt fragment containing a G-box conferred a constitutive expression pattern. Previous studies have indicated that a 955 nt fragment upstream of the translation start site of the *βC1* gene from TYLCCNB is a phloem-specific promoter [14]. Sequence analysis showed that the two putative promoters encompassing the entire non-coding region upstream of the *βC1* open reading frame of MYVB and TYLCCNB shared only 42% nucleotide sequence identity. An ASL box and a TATGAAC motif, which are thought to be responsible for the phloem-specific expression [30], were absent in the promoter region of MYVB. It can be speculated that sequence differences result in the different tissue expression patterns driven by betasatellite promoters.

In conclusion, the MYVB *βC1* promoter directs a constitutive expression pattern in tobacco plants and might be suitable for special plant genetic engineering studies of low-level gene expression.

## Methods

### Construction of plant expression vectors

A series of primers (Table 1) designed according to the MYVB genome (GenBank accession no. AJ421482), were used to amplify the putative promoter region of the *βC1* gene using the previously constructed MYVB infectious clone as the template [15]. The PCR-amplified fragments were cloned into the pGEM-T easy vector (Promega) for sequencing and then digested individually with *Hind*III/*Bam*HI or *Eco*RI/*Sac*I restriction enzymes. The resulting fragments were inserted into the corresponding sites within the binary vector pINT121 or pCHF3: GFP to replace the original CaMV 35S promoter, producing the following expression constructs: pβC1, pβC1-418, pβC1-389, pβC1-267, pβC1-214 and pβC1-138 (Figure 1). The plasmid pINT121 or pCHF3:GFP, in which the reporter gene is driven by the 35S promoter, was used as a positive control. For the negative control, pBINGUS (consisting of the *GUSNos* fragment excised from the *Bam*HI/*Eco*RI sites of pINT121 and inserted into the *Bam*HI/*Eco*RI sites of the pBINPLUS vector) and pCHF3 without the *GFP* gene were used, respectively.

**Table 1:** Sequences of primers used for the PCRs

**Primers**	**Sequences (5'-3')**	**Underlined restriction site**	**Position on MYVB**
Y47βp138-F	GAATTCAAGCTTCTCCATCATATCTTTAAAGT	*Eco*RI, *Hind*III	690-671
Y47βp214-F	GAATTCAAGCTTCTCATGTGTACGTATATATACG	*Eco*RI, *Hind*III	766-745
Y47βp267-F	GAATTCAAGCTTCGTTTTCTTTCCTTTTCAGTTCAG	*Eco*RI, *Hind*III	819-796
Y47βp389-F	GAATTCAAGCTTCAAACGACATCGTTTTCGAGTT	*Eco*RI, *Hind*III	941-920
Y47βp418-F	GAATTCAAGCTTTTATTATTTTTTTCTTTTTTTCTTC	*Eco*RI, *Hind*III	971-947
Y47βp-F	GAATTCAAGCTTTTTCAAATAACAGATCATCAAG	*Eco*RI, *Hind*III	195-174
Y47βp-R	GAGCTCGGATCCCTTGCTTGTGTGATGGAAGTG	*Sac*I, *Bam*HI	553-573
Y47βΔUTR-F	*TTTTCTTTTT*CTCAAACGACATCGTTTTCG	–	962-953 plus 943-924
Y47βΔUTR-R	*GTCGTTTGAG*AAAAAGAAAAAAATAATAATGGGG	–	934-943 plus 953-976
β01	GTAGGTACCACTACGCTACGCAGCAGCC	*Kpn*I	1290-1308
β02	AGTGGTACCTACCCTCCCAGGGGTACAC	*Kpn*I	1283-1265
β03	GTAGAAACCACTACGCTACGCAGCAGCC	–	1290-1308

The 5′UTR Py-rich stretch motif (βC1ΔUTR) was deleted from the entire non-coding region of the *βC1* gene using an overlap-extension PCR strategy [27]. Two independent PCRs were conducted with primer pairs, Y47βp-F/Y47βΔUTR-R and Y47βΔUTR-F/Y47βp-R and PCR products were subsequently added to the standard PCR system and the flanked primer pair Y47βp-F/Y47βp-R was added to amplify the completed βC1ΔUTR. The resulting fragments were digested with *Hind*III/*Bam*HI and inserted into the corresponding sites within the binary vector pINT121 to produce the expression construct, pβC1ΔUTR. Using the same overlap-extension PCR strategy, the full-length MYVB sequence with deletion of the 5′UTR Py-rich stretch (MYVBΔUTR) was obtained. An infectious clone containing MYVBΔUTR was produced as described in [15]. The complete monomeric sequence of MYVBΔUTR was amplified using primers β01/β02. The fragment was then inserted into the pGEM-T easy vector (Promega) to produce the clone, pGEM-MYVBΔUTR. Subsequently, another copy of the complete MYVBΔUTR sequence was amplified using primers β03/β02 to produce pGEM-MYVBΔUTR′. The pGEM-MYVBΔUTR clone was digested with *Kpn*I and inserted into the unique *Kpn*I site of pGEM-MYVBΔUTR′ to produce pGEM-2MYVBΔUTR. Then pGEM-2MYVBΔUTR was digested with *Eco*RI and inserted into the binary vector pBINPLUS to produce pBIN-2MYVBΔUTR, which contains a tandem dimeric repeat of MYVBΔUTR molecules. Infectious clones of MYVV and MYVB were produced previously [15].

Expression vectors were introduced individually into *Agrobacterium tumefaciens* strain EHA105 as described previously [3].

### Transient expression assay

Transient expression analysis by *Agrobacterium*-mediated delivery into plants was carried out as described previously [31]. Three independent experiments were carried out for each construct.

### Fluorometric GFP assay

Leaves of 4 week-old *N*. *benthamiana* plants were infiltrated with the *A*. *tumefaciens* harboring the various expression constructs fused to the *GFP* marker gene. Approximately 64 h after infiltration, 1 cm^2^ leaf fragments were excised and GFP fluorescence was examined in epidermal cells by confocal laser scanning microscopy (CLSM, Leica TCS SP5, Mannheim, Germany).

### Analysis of replication in leaf discs and infected plants

*A*. *tumefaciens* strain EHA105 harboring either helper virus or betasatellite infectious clones was used for infection of *N*. *benthamiana* leaf discs [32] or the whole plants [5] as previously described. Total DNA was extracted using the CTAB method from leaf discs after 6 days or from co-inoculated plants after 30 days. Approximately 10 μg of total DNA was blotted and hybridized with ^32^P-dCTP randomly labeled DNA probes specific for MYVV or MYVB [15]. The band intensities were quantified using Image J software [33].

### Plant transformation

*Agrobacterium*-mediated transformation of *Nicotiana tabacum* leaf discs was conducted according to a previously published procedure [34]. Transformants were selected on Murashige and Skoog medium containing 100 μg/ml kanamycin and 500 μg/ml carbenicillin. Regenerated kanamycin-resistant plants were grown on a rooting medium and then transferred to soil after confirmation by PCR using specific primers for *GUS* gene (5^′^-ATGTTACGTCCTGTAGAAACC-3′/5′-TCATTGTTTGCCTCC CTGC-3′).

### Fluorometric GUS assay and histochemical staining of GUS

*N*. *benthamiana* leaves were sampled 64 h after infiltration and ground in Passive Lysis Buffer (Promega) using a pestle and mortar. Supernatants obtained after centrifugation were used for fluorometric assays. Protein content of the samples was determined by an Eppendorf BioPhotometer (Eppendorf, Hamburg, Germany) using BSA as a standard. Quantitative GUS fluorometric assays were conducted essentially as described by Jefferson et al. [35] and using a Perkin-Elmer LS50B luminescence spectrometer (excitation at 365 nm and emission at 455 nm) to measure the fluorescence of 4-methylumbelliferone (4-MU), which is formed as a result of the cleavage of 4-methylumbelliferyl-β-D-glucuronide (MUG). GUS activity was calculated as the production of 4-MU from MUG in picomoles per minute per microgram of protein. The mean GUS activity from the CaMV 35S promoter of pINT121 was arbitrarily assigned as 100% and used to standardize the activities for all of the other constructs. The resulting data were analyzed using the LSD method of SPSS v12.0 software (SPSS, Chicago, IL, USA).

For the histochemical detection of GUS activity, fresh plant tissue from several transgenic *N*. *tabacum* was incubated for 3 to 12 h in a 5-bromo-4-chloro-3-indolyl β-D-glucuronide staining solution at 37°C as described by Jefferson et al. [35]. The stained samples were cleared by several washes with 70% ethanol and then embedded as described previously [36]. A 11800 Pyramitome (LKB-BROMMA, Stockholm, Sweden) was used for slicing tissue into semi-thin sections. Images of stained sections were photographed with an OLYMPUS BH-2 stereomicroscope (OLYMPUS, Japan).

## Competing interests

The authors declare that they have no conflict of interests.

## Authors' contributions

JZ, XZ, YW and HH performed the experiments. YQ conceived the study. JZ, XZ and YQ wrote the manuscript. All authors read and approved the final manuscript.
